# Bis[2-(2*H*-benzotriazol-2-yl)-4-methylphenolato]palladium(II)

**DOI:** 10.1107/S1600536809016390

**Published:** 2009-05-07

**Authors:** Chen-Yen Tsai, Chia-Her Lin, Bao-Tsan Ko

**Affiliations:** a Department of Chemistry, Chung-Yuan Christian University, Chung-Li 320, Taiwan

## Abstract

In the title complex, [Pd(C_13_H_10_N_3_O)_2_], the Pd^II^ atom is tetra­coordinated by two N atoms and two O atoms from two bidentate 2-(2*H*-benzotriazol-2-yl)-4-methylphenolate ligands, forming a square-planar environment. The asymmetric unit contains one half mol­ecule in which the Pd atom lies on a centre of symmetry.

## Related literature

For background information, see: Deming (1997 ▶); Kricheldorf (2006 ▶); Lin *et al.* (2008 ▶); Peng *et al.* (2008 ▶). For related structures: see: Yang *et al.* (1993 ▶).
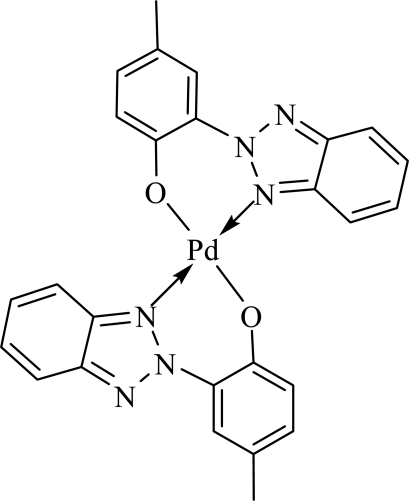


## Experimental

### 

#### Crystal data

[Pd(C_13_H_10_N_3_O)_2_]
*M* *_r_* = 554.88
Monoclinic, 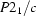

*a* = 12.9768 (7) Å
*b* = 5.6990 (3) Å
*c* = 15.6035 (8) Å
β = 109.287 (3)°
*V* = 1089.19 (10) Å^3^
*Z* = 2
Mo *K*α radiation
μ = 0.89 mm^−1^
*T* = 295 K
0.20 × 0.10 × 0.08 mm

#### Data collection

Bruker APEXII CCD diffractometer
Absorption correction: multi-scan (*SADABS*; Bruker, 2008 ▶) *T*_min_ = 0.842, *T*_max_ = 0.932
9857 measured reflections
2690 independent reflections
1959 reflections with *I* > 2σ(*I*)
*R*_int_ = 0.104

#### Refinement

*R*[*F* ^2^ > 2σ(*F* ^2^)] = 0.031
*wR*(*F* ^2^) = 0.066
*S* = 1.03
2690 reflections
161 parameters
H-atom parameters constrained
Δρ_max_ = 0.42 e Å^−3^
Δρ_min_ = −0.82 e Å^−3^

### 

Data collection: *APEX2* (Bruker, 2008 ▶); cell refinement: *SAINT* (Bruker, 2008 ▶); data reduction: *SAINT*; program(s) used to solve structure: *SHELXS97* (Sheldrick, 2008 ▶); program(s) used to refine structure: *SHELXL97* (Sheldrick, 2008 ▶); molecular graphics: *SHELXTL* (Sheldrick, 2008 ▶); software used to prepare material for publication: *SHELXTL*.

## Supplementary Material

Crystal structure: contains datablocks I, global. DOI: 10.1107/S1600536809016390/rk2144sup1.cif

Structure factors: contains datablocks I. DOI: 10.1107/S1600536809016390/rk2144Isup2.hkl

Additional supplementary materials:  crystallographic information; 3D view; checkCIF report

## Figures and Tables

**Table:** 

Pd—O^i^	1.9676 (15)
Pd—N1	1.9986 (18)

**Table:** 

O^i^—Pd—O	180.0
O^i^—Pd—N1	91.74 (7)
O—Pd—N1	88.26 (7)
N1—Pd—N1^i^	180.0

Symmetry code: (i) 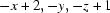.

**Fig. 1.**
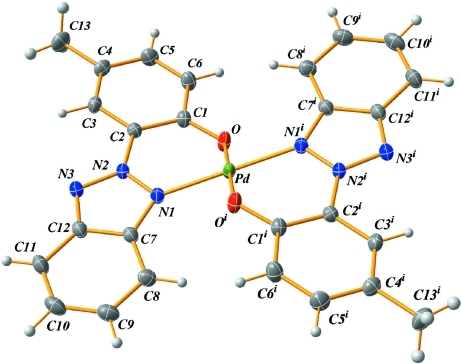
A view of the title molecule I with the atom numbering scheme. Displacement ellipsoids are drawn at the 30% probability level. H atoms are presented as a small spheres of arbitrary radius. Symmetry code: (i) 2-x, -y, 1-z.

**Fig. 2.**
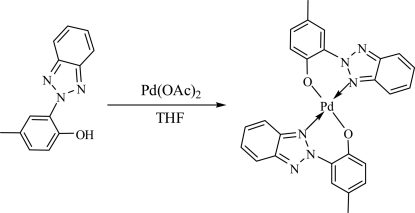
Synthesis pass of title compound I.

## supplementary crystallographic information

### Comment

During the last 2 decades, the synthesis and characterization of polypeptides is an interesting research field that received considerable attentions. The chemical synthesis of high molecular weight poly(α-peptides) can be accomplished by the ring-opening polymerization of α-amino acid *N*-carboxyanhydride (α-*NCA*) initiated by suitable initiators/catalysts. Among these initiators/catalysts, the Ni, Co, Fe, Pd, Pt, Ru, Ir, and Al complexes modified by adequate ligands have been shown to be active initiators/catalysts for *NCA* polymerization (Kricheldorf, 2006). In particular, Deming, (1997), reported that Schiff base ligand containing primary amine complexes of Co, Ni, Pd, and Cu metal ion could efficiently catalyze polymerizations of γ-benzyl *L*-glutamate *N*-carboxyanhydride (*Glu*-*NCA*) to achieve poly(γ-benzyl *L*-glutamate). Recently, Peng *et al.*, 2008, has also reported the Pt complex supported by amido-sulfonamidate ligand and this complex has been demonstrated as efficient initiators for living ROP of α-*NCA*. Most recently, we have successfully synthesized and structural characterized a *N,N',O*-tridentate Schiff base of Cu(II) complex (Lin *et al.*, 2008). We report herein the synthesis and crystal structure of *N,O*-bidentate benzotriazol-phenolate ligands incorporated Pd^II^ complex (I), a potential catalyst for chemical synthesis of poly(peptides) (Scheme 1).

The solid structure of (I) reveals a monomeric Pd^II^ complex (Fig. 1) containing two six-member rings coordinated from these two *N,O*-bidentate benzotriazol-phenolate ligands. It was found that the asymmetric unit has one half of molecule in which the Pd atom lies on a centre of symmetry. The Pd atom is tetra-coordinated with a normal square planar environment in which two N atoms and two O atoms are coplanar. The two N atoms and two O atoms around Pd atom are trans to each other with bond angle of O–Pd–N1 of 91.74 (7)°. The distances between the Pd atom and O and N1 are 1.9676 (15)Å, 1.9986 (18)Å, respectively. These bond distances of Pd–O and Pd–N1 are around 0.1 Å shorter to those found in the other Schiff base Pd^II^ complexes (Yang *et al.*,1993). The bond distance of imine bond, C7–N1 of the benzotriazol group is 1.359 (3)Å and is 0.01Å longer than the other imine bond, C12–N3 (1.348 (3)Å). This is probably due to the existing coordination bond of the former nitrogen, N1.

### Experimental

The title complex was synthesized by the following procedures (Fig. 2): 2-(2*H*-benzotriazol-2-yl)-4-methylphenol (0.45 g, 2.0 mmol) and Pd(O*Ac*)_2_ (0.22 g, 1.0 mmol) was stirred at ambient temperature in *THF* (25 ml) for 12 h during which a red-orange precipitate formed (yield: 75%). The resulting solids were crystallized from CH_2_Cl_2_ solution to yield red crystals. Anal. calcd for C_26_H_20_N_6_O_2_Pd: C, 56.28; H, 3.63; N, 15.15%. Found: C, 56.13; H, 3.79; N, 15.46%.

### Refinement

The H atoms were placed in idealized positions and constrained to ride on their parent atoms, with C–H = 0.93 and 0.96Å with *U*_iso_(H) = 1.2 and 1.5*U*_eq_(C).

### Crystal data

**Table:**

[Pd(C_13_H_10_N_3_O)_2_]	*F*(000) = 560
*M**_r_* = 554.88	*D*_x_ = 1.692 Mg m^−^^3^
Monoclinic, *P*2_1_/*c*	Mo *K*α radiation, λ = 0.71073 Å
Hall symbol: -P 2ybc	Cell parameters from 3862 reflections
*a* = 12.9768 (7) Å	θ = 2.7–28.2°
*b* = 5.6990 (3) Å	µ = 0.89 mm^−^^1^
*c* = 15.6035 (8) Å	*T* = 295 K
β = 109.287 (3)°	Columnar, red
*V* = 1089.19 (10) Å^3^	0.20 × 0.10 × 0.08 mm
*Z* = 2	

### Data collection

**Table:**

Bruker APEXII CCD diffractometer	2690 independent reflections
Radiation source: fine-focus sealed tube	1959 reflections with *I* > 2σ(*I*)
graphite	*R*_int_ = 0.104
φ and ω scans	θ_max_ = 28.3°, θ_min_ = 1.7°
Absorption correction: multi-scan (*SADABS*; Bruker, 2008)	*h* = −16→17
*T*_min_ = 0.842, *T*_max_ = 0.932	*k* = −7→7
9857 measured reflections	*l* = −20→20

### Refinement

**Table:**

Refinement on *F*^2^	Primary atom site location: structure-invariant direct methods
Least-squares matrix: full	Secondary atom site location: difference Fourier map
*R*[*F*^2^ > 2σ(*F*^2^)] = 0.031	Hydrogen site location: inferred from neighbouring sites
*wR*(*F*^2^) = 0.066	H-atom parameters constrained
*S* = 1.03	*w* = 1/[σ^2^(*F*_o_^2^) + (0.019*P*)^2^] where *P* = (*F*_o_^2^ + 2*F*_c_^2^)/3
2690 reflections	(Δ/σ)_max_ = 0.002
161 parameters	Δρ_max_ = 0.42 e Å^−^^3^
0 restraints	Δρ_min_ = −0.82 e Å^−^^3^

### Special details

**Table:**

Geometry. All s.u.'s (except the s.u. in the dihedral angle between two l.s. planes) are estimated using the full covariance matrix. The cell s.u.'s are taken into account individually in the estimation of s.u.'s in distances, angles and torsion angles; correlations between s.u.'s in cell parameters are only used when they are defined by crystal symmetry. An approximate (isotropic) treatment of cell s.u.'s is used for estimating s.u.'s involving l.s. planes.
Refinement. Refinement of F^2^ against ALL reflections. The weighted R-factor wR and goodness of fit S are based on F^2^, conventional R-factors R are based on F, with F set to zero for negative F^2^. The threshold expression of F^2^ > 2σ(F^2^) is used only for calculating R-factors(gt) etc. and is not relevant to the choice of reflections for refinement. R-factors based on F^2^ are statistically about twice as large as those based on F, and R-factors based on ALL data will be even larger.

### Fractional atomic coordinates and isotropic or equivalent isotropic displacement parameters (Å^2^)

**Table:**

	*x*	*y*	*z*	*U*_iso_*/*U*_eq_	
Pd	1.0000	0.0000	0.5000	0.02562 (9)	
O	0.98900 (13)	0.1401 (3)	0.38204 (11)	0.0359 (4)	
N1	0.84675 (14)	−0.1144 (3)	0.44409 (13)	0.0284 (4)	
N2	0.76972 (15)	−0.0006 (3)	0.37741 (13)	0.0270 (4)	
N3	0.66977 (16)	−0.0858 (3)	0.35839 (14)	0.0328 (5)	
C1	0.89890 (19)	0.2481 (4)	0.33261 (16)	0.0309 (5)	
C2	0.79138 (18)	0.1917 (4)	0.32782 (15)	0.0280 (5)	
C3	0.70175 (19)	0.3162 (4)	0.27150 (16)	0.0324 (5)	
H3B	0.6319	0.2736	0.2698	0.039*	
C4	0.7139 (2)	0.4982 (4)	0.21906 (17)	0.0352 (5)	
C5	0.8191 (2)	0.5568 (4)	0.22219 (18)	0.0406 (7)	
H5A	0.8293	0.6805	0.1870	0.049*	
C6	0.9083 (2)	0.4351 (4)	0.27641 (18)	0.0392 (6)	
H6A	0.9773	0.4775	0.2760	0.047*	
C7	0.79189 (19)	−0.2925 (4)	0.46805 (16)	0.0301 (5)	
C8	0.8287 (2)	−0.4786 (4)	0.52925 (18)	0.0375 (6)	
H8A	0.9021	−0.4980	0.5630	0.045*	
C9	0.7499 (2)	−0.6305 (4)	0.53637 (18)	0.0429 (7)	
H9A	0.7707	−0.7559	0.5766	0.051*	
C10	0.6391 (2)	−0.6036 (5)	0.48527 (19)	0.0463 (7)	
H10A	0.5890	−0.7107	0.4933	0.056*	
C11	0.6026 (2)	−0.4273 (4)	0.4248 (2)	0.0408 (6)	
H11A	0.5291	−0.4116	0.3908	0.049*	
C12	0.68220 (19)	−0.2680 (4)	0.41576 (17)	0.0328 (5)	
C13	0.6164 (2)	0.6324 (5)	0.15814 (19)	0.0511 (7)	
H13A	0.5514	0.5803	0.1690	0.077*	
H13B	0.6094	0.6049	0.0958	0.077*	
H13C	0.6266	0.7971	0.1711	0.077*	

### Atomic displacement parameters (Å^2^)

**Table:**

	*U*^11^	*U*^22^	*U*^33^	*U*^12^	*U*^13^	*U*^23^
Pd	0.01933 (15)	0.02899 (11)	0.02833 (15)	0.00099 (10)	0.00758 (11)	0.00236 (11)
O	0.0225 (9)	0.0518 (10)	0.0342 (10)	0.0034 (7)	0.0102 (8)	0.0126 (8)
N1	0.0228 (11)	0.0311 (9)	0.0307 (11)	0.0003 (8)	0.0080 (9)	0.0017 (8)
N2	0.0196 (10)	0.0309 (8)	0.0289 (11)	−0.0003 (8)	0.0057 (9)	−0.0005 (9)
N3	0.0215 (12)	0.0359 (9)	0.0387 (12)	−0.0033 (8)	0.0070 (10)	−0.0023 (9)
C1	0.0281 (14)	0.0388 (11)	0.0259 (13)	−0.0002 (10)	0.0089 (11)	−0.0002 (10)
C2	0.0285 (14)	0.0302 (10)	0.0255 (13)	−0.0014 (9)	0.0090 (11)	−0.0011 (9)
C3	0.0254 (14)	0.0373 (11)	0.0332 (14)	0.0034 (9)	0.0079 (12)	−0.0021 (10)
C4	0.0361 (15)	0.0391 (11)	0.0264 (13)	0.0074 (11)	0.0049 (11)	0.0006 (11)
C5	0.0445 (18)	0.0419 (14)	0.0335 (15)	−0.0010 (10)	0.0107 (14)	0.0095 (10)
C6	0.0302 (15)	0.0493 (13)	0.0368 (16)	−0.0050 (10)	0.0093 (13)	0.0086 (11)
C7	0.0287 (14)	0.0312 (10)	0.0320 (14)	−0.0044 (9)	0.0124 (12)	−0.0045 (9)
C8	0.0385 (16)	0.0358 (12)	0.0359 (15)	−0.0038 (10)	0.0093 (13)	0.0021 (11)
C9	0.057 (2)	0.0341 (12)	0.0400 (16)	−0.0080 (11)	0.0194 (15)	0.0011 (11)
C10	0.053 (2)	0.0425 (13)	0.0505 (19)	−0.0198 (13)	0.0266 (16)	−0.0053 (13)
C11	0.0329 (16)	0.0444 (12)	0.0467 (18)	−0.0132 (11)	0.0154 (14)	−0.0069 (12)
C12	0.0284 (14)	0.0349 (11)	0.0365 (14)	−0.0046 (9)	0.0124 (12)	−0.0067 (10)
C13	0.0457 (18)	0.0549 (16)	0.0448 (18)	0.0142 (13)	0.0041 (15)	0.0105 (13)

### Geometric parameters (Å, °)

**Table:**

Pd—O^i^	1.9676 (15)	C5—C6	1.375 (4)
Pd—O	1.9677 (15)	C5—H5A	0.9300
Pd—N1	1.9986 (18)	C6—H6A	0.9300
Pd—N1^i^	1.9986 (18)	C7—C12	1.394 (3)
O—C1	1.321 (3)	C7—C8	1.401 (3)
N1—N2	1.347 (3)	C8—C9	1.372 (3)
N1—C7	1.361 (3)	C8—H8A	0.9300
N2—N3	1.324 (3)	C9—C10	1.403 (4)
N2—C2	1.422 (3)	C9—H9A	0.9300
N3—C12	1.346 (3)	C10—C11	1.353 (4)
C1—C2	1.410 (3)	C10—H10A	0.9300
C1—C6	1.410 (3)	C11—C12	1.417 (3)
C2—C3	1.398 (3)	C11—H11A	0.9300
C3—C4	1.362 (3)	C13—H13A	0.9600
C3—H3B	0.9300	C13—H13B	0.9600
C4—C5	1.391 (4)	C13—H13C	0.9600
C4—C13	1.515 (3)		
			
O^i^—Pd—O	180.0	C4—C5—H5A	119.4
O^i^—Pd—N1	91.74 (7)	C5—C6—C1	122.4 (2)
O—Pd—N1	88.26 (7)	C5—C6—H6A	118.8
O^i^—Pd—N1^i^	88.27 (7)	C1—C6—H6A	118.8
O—Pd—N1^i^	91.73 (7)	N1—C7—C12	106.86 (19)
N1—Pd—N1^i^	180.0	N1—C7—C8	131.3 (2)
C1—O—Pd	120.80 (13)	C12—C7—C8	121.8 (2)
N2—N1—C7	104.42 (18)	C9—C8—C7	115.9 (3)
N2—N1—Pd	123.90 (14)	C9—C8—H8A	122.0
C7—N1—Pd	131.19 (16)	C7—C8—H8A	122.0
N3—N2—N1	114.76 (18)	C8—C9—C10	122.4 (2)
N3—N2—C2	121.06 (19)	C8—C9—H9A	118.8
N1—N2—C2	124.15 (18)	C10—C9—H9A	118.8
N2—N3—C12	103.9 (2)	C11—C10—C9	122.3 (2)
O—C1—C2	126.5 (2)	C11—C10—H10A	118.8
O—C1—C6	118.3 (2)	C9—C10—H10A	118.8
C2—C1—C6	115.3 (2)	C10—C11—C12	116.5 (3)
C3—C2—C1	121.4 (2)	C10—C11—H11A	121.7
C3—C2—N2	117.4 (2)	C12—C11—H11A	121.7
C1—C2—N2	121.1 (2)	N3—C12—C7	110.0 (2)
C4—C3—C2	121.8 (2)	N3—C12—C11	129.0 (2)
C4—C3—H3B	119.1	C7—C12—C11	121.0 (2)
C2—C3—H3B	119.1	C4—C13—H13A	109.5
C3—C4—C5	117.9 (2)	C4—C13—H13B	109.5
C3—C4—C13	121.6 (2)	H13A—C13—H13B	109.5
C5—C4—C13	120.5 (2)	C4—C13—H13C	109.5
C6—C5—C4	121.2 (2)	H13A—C13—H13C	109.5
C6—C5—H5A	119.4	H13B—C13—H13C	109.5

Symmetry codes: (i) −*x*+2, −*y*, −*z*+1.
